# Salvage surgery for neck residue or recurrence of nasopharyngeal carcinoma after primary radiotherapy: options of surgical methods and regions

**DOI:** 10.1186/s12957-016-0822-8

**Published:** 2016-03-25

**Authors:** Sheng-ye Wang, Jian-lin Lou, Jianxiang Chen, Su-zhan Zhang, Liang Guo

**Affiliations:** Department of Oncology, Second Affiliated Hospital, School of Medicine, Zhejiang University, 88# Jiefang Road, Hangzhou, 310009 China; Department of Radiation Oncology, Zhejiang Cancer Hospital, Hangzhou, 310022 People’s Republic of China; Department of Head and Neck Surgery, Zhejiang Cancer Hospital, Hangzhou, 310022 People’s Republic of China

**Keywords:** Nasopharyngeal carcinoma, Salvage surgery, Residual, Recurrence, Neck dissection

## Abstract

**Background:**

Salvage surgery has been recommended as the approach of choice for neck residue or recurrence of nasopharyngeal carcinoma (NPC) after primary radiotherapy (RT). This study aimed to assess the outcome and prognostic factors, options for different surgical methods, and the extent of neck dissection (ND) for patients.

**Methods:**

NPC patients who had undergone RT and received salvage surgery for neck residue or recurrence from January 2001 to December 2011 were retrospectively analyzed. The overall survival (OS) rate was calculated by Kaplan-Meier method, and prognostic factors were determined by log-rank test and Cox regression analysis.

**Results:**

In 153 cases, 96 cases have level I dissections. The metastasis rate was 20/153 (13.07 %) for level I metastasis and 7/153 (4.58 %) for parotid gland cases. The 3- and 5-year OS rate was 57.2 and 40.6 %, respectively, and median survival time was 49 months. By univariate analysis, the age, rN staging, size of lymph nodes (LN), extra-capsular spread (ECS), and surgical procedure were significant prognostic factors. By multivariable analysis, the age, rN staging, and size of LN were significant prognostic factors.

**Conclusions:**

Salvage surgery is effective for neck failure of NPC after primary treatment, but patients with age >50 years, stage rN3, or LN >6 cm have poor prognosis.

## Background

Nasopharyngeal carcinoma (NPC) is a prevalent disease in southern China with unique epidemiology, pathology, and treatment outcome [1]. Radiotherapy (RT) or chemoradiotherapy (CRT) has been standard treatment for newly diagnosed NPC [2, 3]. But for residual or recurrent NPC, surgery is needed [4]. NPC is easy to metastasize to cervical lymph nodes (LN), and the incidence of residual or recurrent NPC is 4.6–18 % [5–7]. Neck dissection (ND) has been proposed for poor outcomes after primary NPC treatment.

After primary treatment, regional neck disease of NPC is prone to extra-capsular spread (ECS). Therefore, radical neck dissection (RND) is proposed for the control of regional NPC [8–11]. Despite the development of surgical technique, the surgical traumas and complications remain. Currently, the preservation of the internal jugular vein, sternocleidomastoid muscle, and/or spinal accessory nerve and even selective cervical neck dissections to protect external jugular vein and cervical plexus have been widely performed in head and neck squamous carcinoma and thyroid cancer. Modified radical neck dissection (MRND) for preserving the internal jugular vein, sternocleidomastoid muscle, and/or spinal accessory nerve is technically feasible. However, whether MRND could improve survival rate of patients and minimize surgical morbidity remains unclear.

In this study, we retrospectively reviewed the medical records of patients with neck regional failure after primary NPC treatment to assess different surgical methods and the extent of neck dissection for neck residue or recurrence of NPC after primary RT. We also analyzed prognostic factors on treatment outcomes such as regional control and overall survival.

## Methods

### Patients

The study protocol was approved by Ethics Committee of Zhejiang Cancer Hospital. This retrospective study included 153 patients, who had previously undergone RT with or without chemotherapy, had untreated neck residue or recurrence of NPC with no evidence of local disease and distant metastases, and then were treated with salvage surgery. All patients underwent neck dissection at the Department of Head and Neck Surgery, Zhejiang Cancer Hospital, from January 2001 to December 2011. There were 128 males and 25 females. The median age was 49 years (ranged from 18 to 76 years) at the time of treating for neck regional recurrence or residue.

**Fig. 1:**
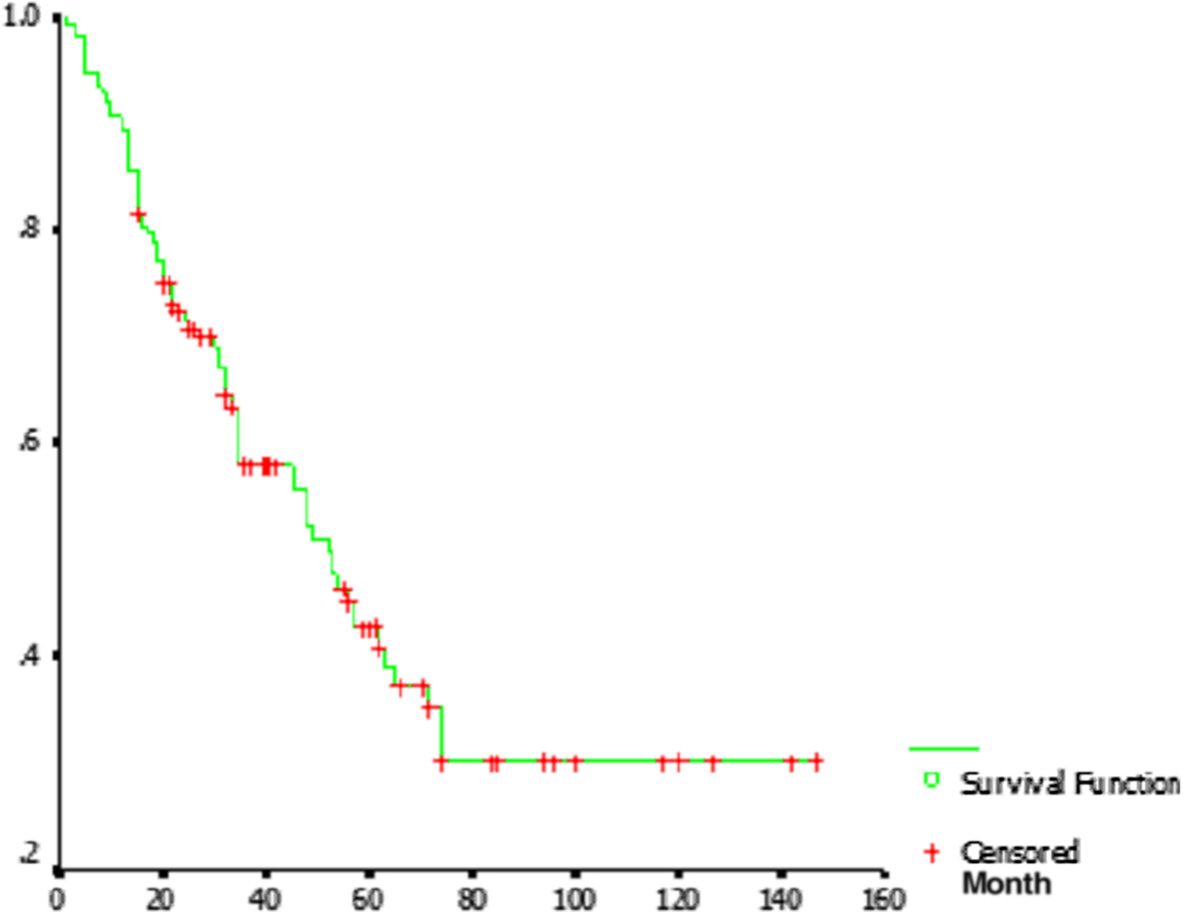
The survival curve of patients undergoing salvage surgery with the diagnosis of neck recurrence or residue of nasopharyngeal carcinoma. *X axial*: survival time (months); *Y axial*: cumulative survival rate

NPC patients were treated with standard RT, which consisted of 65–78 Gy to primary tumor, 60–70 Gy to involved LN, 50 Gy to uninvolved neck, and daily dose 1.8–2.0 Gy/5 days a week. One hundred thirty-one NPC patients got additional chemotherapy. Regional NPC recurrence was confirmed by fine-needle aspiration (FNA) or open biopsy. All neck metastases were pathologically identified after the surgery. Before receiving salvage treatment for regional failure, all patients received complete physical examination, nasopharyngoscopy and biopsy of suspicious nasopharyngeal lesions, magnetic resonance imaging (MRI) scans and/or computed tomography (CT) of nasopharynx and neck, CT or X-ray of chest, color Doppler ultrasound of abdominal and neck, and bone scanning were carried out to exclude local disease and distant metastases.

### Tumor characteristics

Recurrent disease was defined as the reappearance of cervical LN 3 months after initial complete regression, and residual disease was defined as the lymph nodes without complete regression by 3 months after primary therapy as described previously [9]. The group with recurrent nodal disease consisted of 105 patients (68.6 %), while the group with residual nodal disease consisted of 48 patients (31.4 %). The interval between the finishing time of RT/CRT and cervical relapse was from 4 to 192 months (median, 30 months).

Among the 153 patients, 109 patients were stage rN1, 17 were stage rN2, and 27 were stage rN3, according to the 2002 American Joint Committee on Cancer (AJCC) Classification staging system [12]. Sixty-eight cases were left, 68 cases were right, and 17 cases were bilateral. According to the lymph nodal sites, level II was the most common site of nodal involvement, followed by levels V, III, IV, and I. In level II, 122 cases (79.7 %) had LN metastasis. Twenty cases (13.1 %) had LN metastasis in level I and 7 cases (4.6 %) had parotid gland metastasis. Pathological analysis of surgical specimens showed that 111 tumors (72.5 %) had ECS.

### Follow-up

The patients were followed up by visiting hospital for evaluation of recurrence every 3 months. They were followed up by phone call or letter for survival if they failed to visit the hospital. The starting point of the follow-up period was defined as the completion of salvage treatment for regional recurrence or residue, while the end point was defined as when the patient died or June 2013.

### Statistical analysis

Data were analyzed by using SPSS 16.0 software (SPSS Inc, Chicago IL, USA). Overall survival rate was calculated by Kaplan-Meier method. The differences between groups were analyzed by chi-square test, and the statistical significance of regional control was determined using logistic regression. *P* < 0.05 was considered significant difference.

## Results

### Modality of salvage treatment

All 153 patients received neck dissection as salvage surgery for neck regional failure after primary NPC treatment at the Department of Head and Neck Surgery, Zhejiang Cancer Hospital. Seventeen patients received bilateral ND simultaneously, so totally, 170 cases had ND on the lateral basis. The classification of ND was as follows: 84 laterals received ND for levels I–V, 44 for levels II–V, 17 for levels I–III, 9 for cervical local excision, 5 for levels I–II, 2 for levels II–VI, 2 for levels III–V, 3 for parotidectomy, 2 for totally parotidectomy + levels I–II, 2 for levels I–V + parotidectomy. Seven patients were treated with the removal of ipsilateral parotid LN and total or partial parotidectomy. The surgical procedures taken were as follows: 106 patients received MRND (including SND in 28, local excision in 9, and parotidectomy in 3), 48 patients received RND, and 16 patients received enlarged radical neck dissection (ERND). ND was performed via different approaches based on the characteristics of the diseases (Table 1).

**Table 1:** Characteristics of NPC undergoing MRND and RND/ERND

Characteristic	Cases observed	Surgical procedure		*χ* ^*2*^ value	*P*
		MRND (89)	RND/ERND (64)		
Age (year)					
≤50	90	55	35		
>50	63	34	29	0.777	0.378
Gender					
Male	128	74	54		
Female	25	15	10	0.041	0.839
Status of LN					
Recurrence	105	54	51		
Residue	48	35	13	6.251	0.012
rN stage					
N1	109	79	30		
N2	17	8	9		
N3	27	2	25	38.625	0.000
Size of LN					
≤3 cm	80	68	12		
3~6 cm	47	20	27		
>6 cm	26	1	25	59.911	0.000
ECS					
Yes	111	47	64		
No	42	42	0	41.630	0.000

For MRND, 64 cases had total preservation of the internal jugular vein, sternocleidomastoid muscle, and spinal accessory nerve, 18 cases had preservation of the internal jugular vein and spinal accessory nerve, 5 cases had preservation of the internal jugular vein and sternocleidomastoid muscle, 16 cases had preservation of the internal jugular vein, and 3 cases had preservation of the spinal accessory nerve. For ERND, 12 cases had the skin involved, 4 cases had the phrenic nerve involved, 2 cases had the carotid artery involved, 2 cases had the vagus nerve involved, 2 cases had the submaxillary involved, and 1 case had the hypoglossal nerve involved. Twenty-five cases underwent pedicled pectoralis major muscle flap surgery. Among 153 patients, 10 received postoperative re-RT and 23 received combination of chemotherapy.

### Survival analysis

During the follow-up period of 2 to 147 months (median 29 months), 74 of 153 patients were alive, while 79 patients died. Kaplan-Meier survival analysis indicated that total 3-year OS was 57.2 % and 5-year OS was 40.6 %. The median survival time (MST) was 49 months (Fig. 1). Univariate analysis showed that the age, rN staging, size of LN, ECS, and surgical procedure were significant factors affecting the survival rate (*P* < 0.05). Cox analysis revealed that the age, rN staging, and size of LN were the main factors affecting the survival rate (Tables 2 and 3).

**Table 2:** Univariate analysis of prognostic factors for OS of 153 patients

Parameter	Cases observed	OS		*χ* ^*2*^ value	*P*
		3-year (%)	5-year (%)		
Age (year)					
≤50	90	71.5	55.5		
>50	63	39.4	22.8	13.360	0.000
Gender					
Male	128	57.1	42.9		
Female	25	59.2	23.7	1.401	0.237
Status of LN					
Recurrence	105	59.5	43.1		
Residue	48	50.9	33.9	1.642	0.200
rN stage					
N1	109	67.7	47.0		
N2	17	44.9	36.0		
N3	27	20.7	15.6	40.152	0.000
Size of LN					
≤3 cm	80	67.5	52.4		
3~6 cm	47	58.9	34.7		
>6 cm	26	21.5	16.2	40.012	0.000
Bilateral of LN					
Yes	17	44.9	36.0		
NO	136	58.5	40.9	0.005	0.943
ECS					
Yes	111	51.8	35.7		
No	42	71.3	54.8	6.189	0.013
Level 1 or parotid metastasis					
Yes	27	54.8	38.4		
No	126	57.6	41.0	0.085	0.771
Surgical procedure					
MRND	89	66.3	47.9		
RND/ERND	64	44.3	30.5	11.823	0.001

**Table 3:** Cox regression model of multivariable analysis for OS

Factor	*β*	SE	Wald	*P*	Exp (*β*)
Age	0.809	0.232	12.193	0.000	2.245
rN stage	0.449	0.186	5.852	0.016	1.567
Size of LN	0.431	0.203	4.527	0.033	1.539

### Regional control

In follow-up, regional recurrence or failure was noted in 21 (13.7 %) patients: 12 had ipsilateral neck recurrence, 3 had ipsilateral parotid region relapse, 2 had ipsilateral parapharyngeal lymph node relapse, 2 had oral cavity and oropharynx secondary primary carcinoma, 1 had nasopharyngeal carcinoma relapse, and 1 had contralateral neck recurrence. The relapse time was 2 to 38 months (median 12 months).

### Distant metastasis

Twenty-nine patients (19.0 %) developed distant metastasis 6 to 48 months after treatment. Among them, 8 cases survived with tumor, while 2 cases died. The most common site of metastasis was the lung (18), followed by the liver (9), bone (8), abdominal cavity (3), mediastinum (3), and axillary nodes (2).

### Complications

After surgery, there was no surgical mortality in any patients, but several complications were observed as follows: incision infection (6 cases), debridement due to disruption of wound or flap necrosis (5 cases), chylous linkage (4 cases), post-operational bleeding (2 cases), deviated mouth (2 cases), hoarseness (2 cases), bucking (2 cases), Horner’s syndrome (2 cases), injury of hypoglossal nerve (1 case), prosopoplegia (1 case), and induced or aggravated radiation encephalopathy (1 case).

## Discussion

NPC is a common tumor of the head and neck that is prevalent in southern China. RT/CRT rather than surgery is the primary treatment for NPC, due to the anatomical location and the radiosensitivity of the tumor. Despite regional control by RT/CRT, NPC has a tendency to metastasize to cervical LN and the incidence of residual or recurrent NPC is 4.6–18 % [6, 7]. There is still a problem with how to approach failures associated with the first treatment for neck lesion.

Most regional persistent or recurrent disease is technically resectable and is usually salvaged surgically. Thus, salvage surgery is important for the treatment of recurrent nodal disease [8, 9, 13]. Zhang et al. used salvage ND to treat 355 NPC patients who were diagnosed with neck residue or recurrence after RT, and the 3-and 5-year OS was 54.11 and 26.03 %, respectively [14]. In this study, we retrospectively reviewed 153 patients with NPC, the 3-year and 5-year OS was 57.2 and 40.6 %, respectively; and MST was 49 months after salvage surgery for neck residue or recurrence. Furthermore, we determined that the age, rN staging, and size of LN are the factors that affect survival rate. In this cohort, the prognosis of patients with age ≤50 years old was better than patients with age >50 years old. The reason is unknown but may be due to that younger patients are much more apt to intensive treatment.

Currently, there are still no uniform standards on the options of surgical methods and regions for ND. The extent of surgical resection might range from excision of the enlarged node to SND, MRND (removing all the lymphatic tissue and LN in the neck while preserving non-lymphatic structures such as the internal jugular vein, sternocleidomastoid muscle, and/or spinal accessory nerve), and RND to ERND. The type of salvage surgery depends on the pathological aspects of metastatic NPC in the LN. Residual lymph nodes after NPC radiation tend to be highly malignant and invasive and have a high incidence of ECS to the non-lymphatic structure. RND is suggested to be the optimal treatment choice to remove neck disease, and it has become the mainstay of treatment for regional failure of NPC [15–17]. However, RND may impair daily activities of the patients because the sacrifice of the spinal accessory nerve would cause shoulder pain, and the ligation of the internal jugular vein would cause facial swelling [18]. In contrast, MRND gets rid of all lymphatic tissues of the neck but preserves the spinal accessory nerve, sternocleidomastoid muscle, and/or internal jugular vein, reducing surgical morbidity.

In this study, we performed surgery on 153 patients, with 170 cases of cervical dissections on the lateral basis. Among them, 106 cases received MRND. MRND could reduce surgical morbidity and complications such as shoulder pain and facial swelling and thus improve the quality of life of the patients. Meanwhile, RND is the appropriate approach for isolated regional failure of NPC, especially when NPC is advanced or the spinal accessory nerve and internal jugular vein are involved.

There are several complications after NPC cervical radiation, such as tissue fibrosis, muscle atrophy and ischemia of the skin. So we could selectively use myocutaneous flap with vessel pedicle such as pedicled pectoralis major muscle flap in RND after NPC radiotherapy. The merits of this surgery are as follows: rapid postoperative recovery, enough tissue to select, powerful anti-infection ability, easy to close dead space, protecting large vessels such as carotid artery, and avoiding serious complications. In this study, 25 cases had surgery of pedicled pectoralis major muscle flap, and the outcomes were satisfied without flap necrosis after flap transpositions.

Early reports suggest that RND should be adopted when cervical LNs recur after NPC radiotherapy. RND ranges over complete excisions of the whole tissues including the sternocleidomastoid muscle, internal jugular vein, accessory nerve, accessory nerve, submandibular gland, inferior portion of the parotid gland, and peripheral lymphoid tissues. Sham et al. reported distributing characteristics of cervical LNs in NPC; the metastasis rate of levels I, II, III, IV, and V was 12.7, 95.4, 60.2, 15.5, and 44.0 %, respectively. The metastasizing region was mainly focused on level II, and level I had the lowest metastasis rate [19]. Yen et al. reported that the incidence of cervical recurrence of NPC was 81 % at levels II and V, while the relapse rate was much lower for levels I and III, and they suggested that NRD surgery of NPC should mainly concentrate on levels II and IV [10].

In this cohort of 153 cases, level II was the highest nodal metastasis site (79.7 %). Level II should be considered as an important dissected region. Metastasis nodes in levels III to V are always distributed multiregionally, melting together and hardly distinguished by separate level. In our study, 96 cases had level I dissection. The total relapse rate of case at level I was 13.7 %. Seven cases had parotid gland metastasis and 3 cases had ipsilateral parotid gland relapse. Patients with level I metastasis and without level I metastasis showed no significant differences in 3-year and 5-year OS (*P* = 0.7711). So we suggest that level I and parotid metastasis should be paid some attention to, but not be the routine dissected region. Further studies with larger samples are needed to address how different metastasis levels affect the outcome of therapy.The total relapse rate of parotid gland was 6.5 %. We suggest that we should carefully assess the lymph nodes in level I and parotid gland before surgery and selectively dissect level I and parotid gland in some occasions. However, it may enhance difficulty and risk of surgery if we routinely dissect level I and parotid gland. If there are no metastasized LNs in level I after preoperational evaluation, it is unnecessary to have routine dissection in this region. If LN residual or relapsed is detected in parotid region, total parotidectomy and suitable procedure should be operated. If metastasized LNs in the upper neck are close to the tail of parotid, partial parotidectomy should be performed for cervical ND.

## Conclusions

Salvage surgery is effective for neck failure of NPC after primary treatment, but patients with age >50 years or stage rN3 or LN >6 cm have poor prognosis. MRND is suitable for most of cases, even if some of them have ECS. LN in level I and parotid gland do not need neck dissection, but careful examination is necessary.

## Consent

Written informed consent was obtained from the patients for the publication of this report and any accompanying images.
